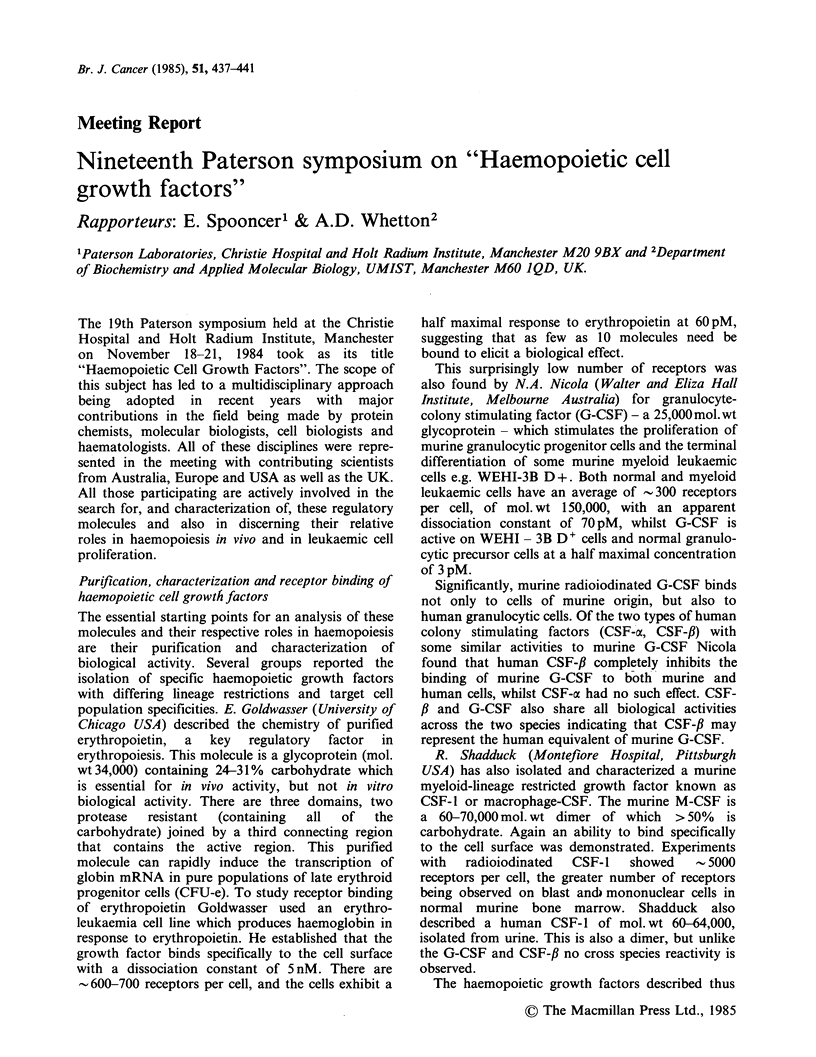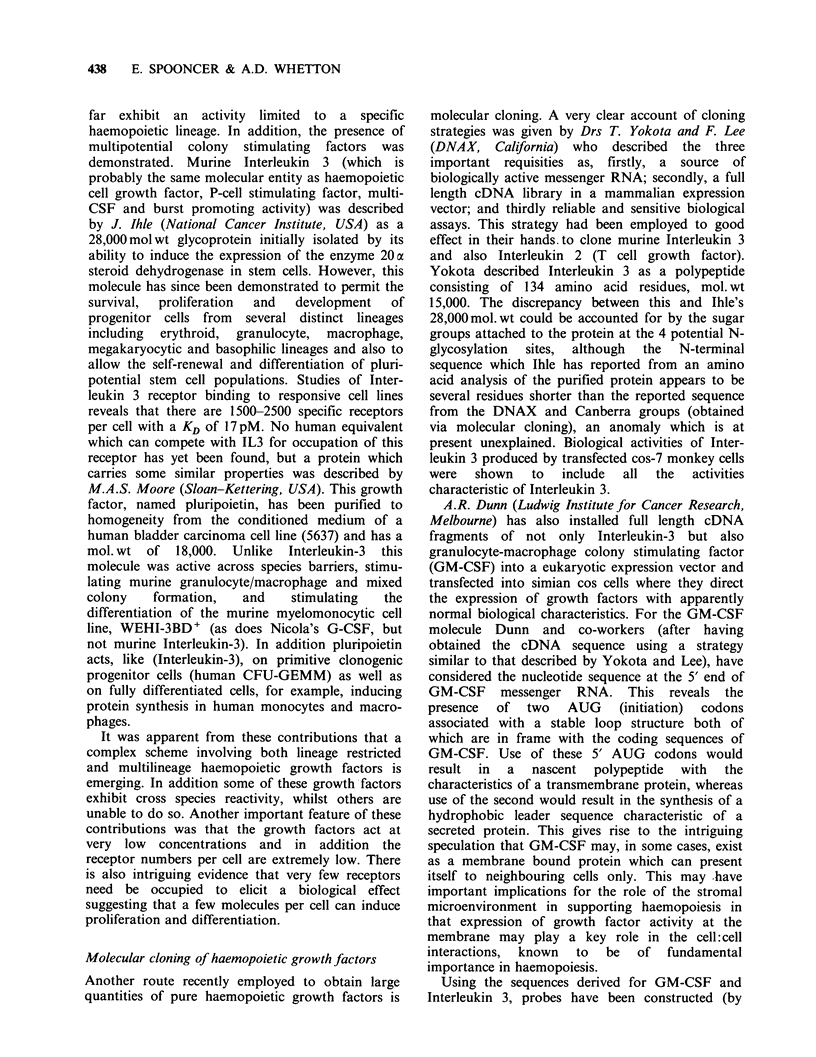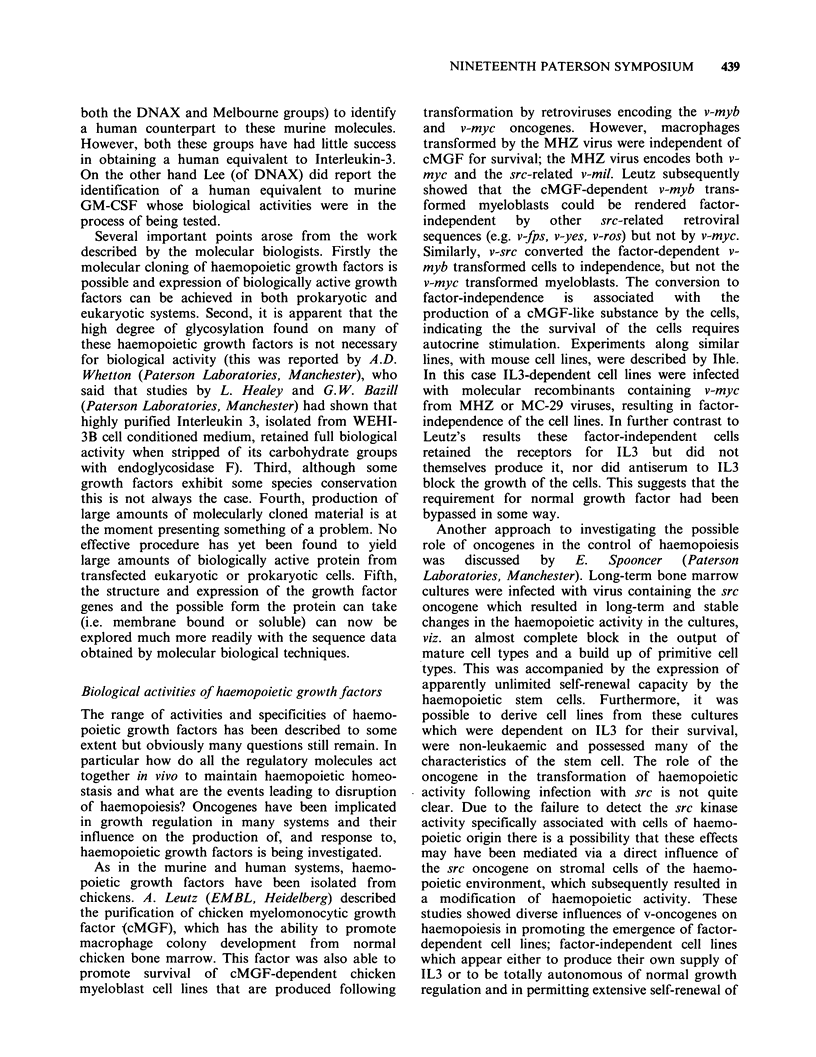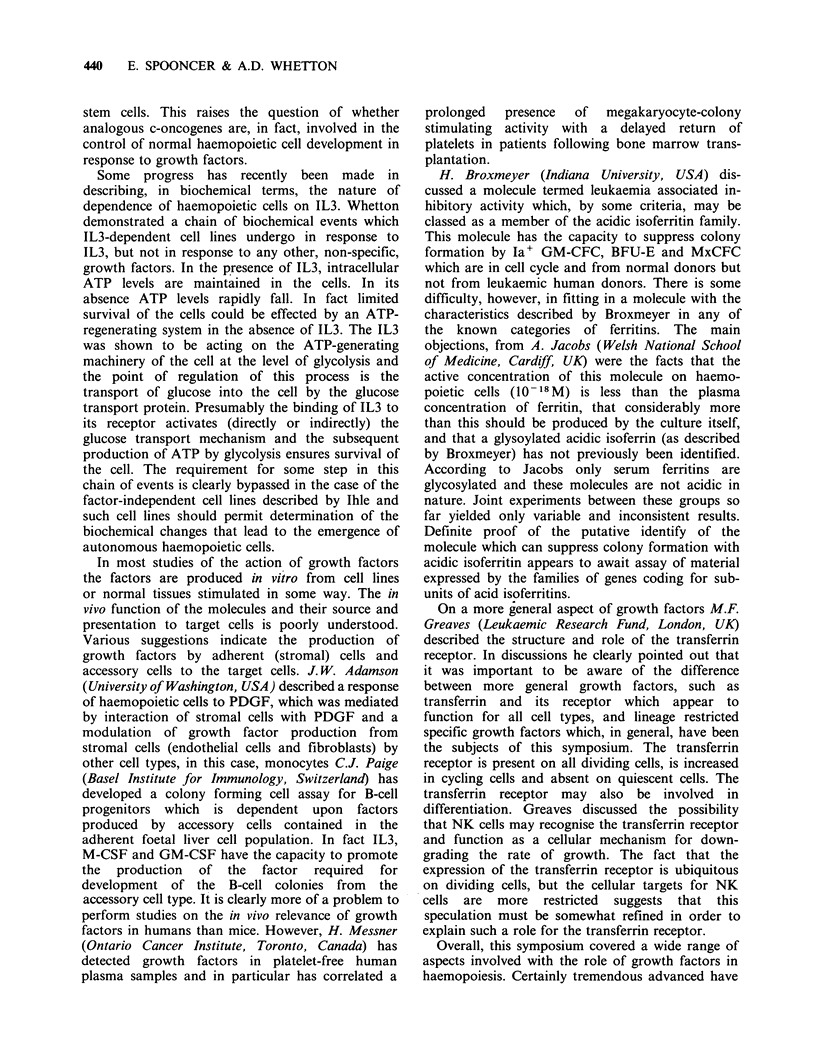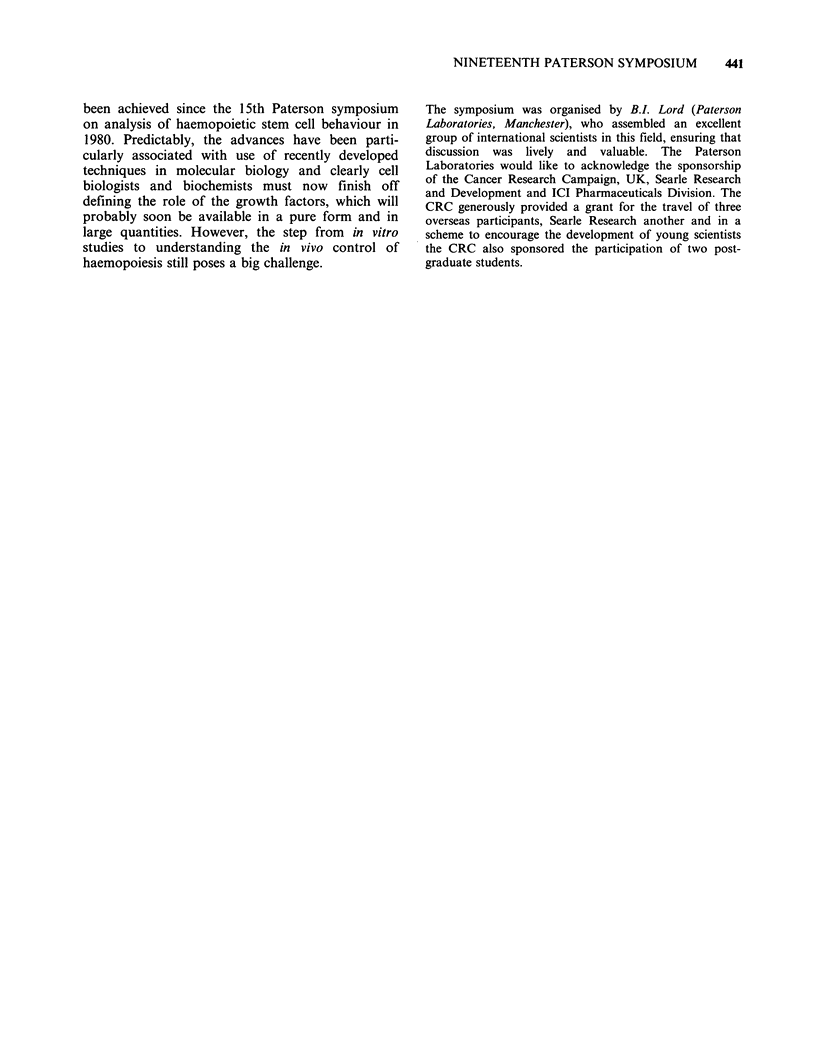# Nineteenth Paterson Symposium on “Haemopoietic Cell Growth Factors”

**Published:** 1985-03

**Authors:** 


*Br. J. Cancer* (1985), **51**, 437–441

### Meeting Report

### Nineteenth Paterson symposium on "Haemopoietic cell growth factors"

*Rapporteurs*: E. Spooncer[1] & A.D. Whetton[2]

[1]*Paterson Laboratories, Christie Hospital and Holt Radium Institute, Manchester M20 9BX and* [2]*Department of Biochemistry and Applied Molecular Biology, UMIST, Manchester M60 1QD, UK.*

The 19th Paterson symposium held at the Christie Hospital and Holt Radium Institute, Manchester on November 18–21, 1984 took as its title "Haemopoietic Cell Growth Factors". The scope of this subject has led to a multidisciplinary approach being adopted in recent years with major contributions in the field being made by protein chemists, molecular biologists, cell biologists and haematologists. All of these disciplines were represented in the meeting with contributing scientists from Australia, Europe and USA as well as the UK. All those participating are actively involved in the search for, and characterization of, these regulatory molecules and also in discerning their relative roles in haemopoiesis *in vivo* and in leukaemic cell proliferation.

*Purification, characterization and receptor binding of haemopoietic cell growth factors*

The essential starting points for an analysis of these molecules and their respective roles in haemopoiesis are their purification and characterization of biological activity. Several groups reported the isolation of specific haemopoietic growth factors with differing lineage restrictions and target cell population specificities. *E. Goldwasser* (*University of Chicago USA*) described the chemistry of purified erythropoietin, a key regulatory factor in erythropoiesis. This molecule is a glycoprotein (mol. wt 34,000) containing 24–31% carbohydrate which is essential for *in vivo* activity, but not *in vitro* biological activity. There are three domains, two protease resistant (containing all of the carbohydrate) joined by a third connecting region that contains the active region. This purified molecule can rapidly induce the transcription of globin mRNA in pure populations of late erythroid progenitor cells (CFU-e). To study receptor binding of erythropoietin Goldwasser used an erythroleukaemia cell line which produces haemoglobin in response to erythropoietin. He established that the growth factor binds specifically to the cell surface with a dissociation constant of 5 nM. There are ~600–700 receptors per cell, and the cells exhibit a half maximal response to erythropoietin at 60 pM, suggesting that as few as 10 molecules need be bound to elicit a biological effect.

This surprisingly low number of receptors was also found by *N.A. Nicola* (*Walter and Eliza Hall Institute, Melbourne Australia*) for granulocyte-colony stimulating factor (G-CSF) – a 25,000 mol. wt glycoprotein – which stimulates the proliferation of murine granulocytic progenitor cells and the terminal differentiation of some murine myeloid leukaemic cells e.g. WEHI-3B D+. Both normal and myeloid leukaemic cells have an average of ~300 receptors per cell, of mol. wt 150,000, with an apparent dissociation constant of 70 pM, whilst G-CSF is active on WEHI – 3B $D^+$ cells and normal granulocytic precursor cells at a half maximal concentration of 3 pM.

Significantly, murine radioiodinated G-CSF binds not only to cells of murine origin, but also to human granulocytic cells. Of the two types of human colony stimulating factors (CSF-α, CSF-β) with some similar activities to murine G-CSF Nicola found that human CSF-β completely inhibits the binding of murine G-CSF to both murine and human cells, whilst CSF-α had no such effect. CSF-β and G-CSF also share all biological activities across the two species indicating that CSF-β may represent the human equivalent of murine G-CSF.

*R. Shadduck* (*Montefiore Hospital, Pittsburgh USA*) has also isolated and characterized a murine myeloid-lineage restricted growth factor known as CSF-1 or macrophage-CSF. The murine M-CSF is a 60–70,000 mol. wt dimer of which >50% is carbohydrate. Again an ability to bind specifically to the cell surface was demonstrated. Experiments with radioiodinated CSF-1 showed ~5000 receptors per cell, the greater number of receptors being observed on blast and mononuclear cells in normal murine bone marrow. Shadduck also described a human CSF-1 of mol. wt 60–64,000, isolated from urine. This is also a dimer, but unlike the G-CSF and CSF-β no cross species reactivity is observed.

The haemopoietic growth factors described thus

© The Macmillan Press Ltd., 1985

far exhibit an activity limited to a specific haemopoietic lineage. In addition, the presence of multipotential colony stimulating factors was demonstrated. Murine Interleukin 3 (which is probably the same molecular entity as haemopoietic cell growth factor, P-cell stimulating factor, multi-CSF and burst promoting activity) was described by *J. Ihle* (*National Cancer Institute, USA*) as a 28,000 mol wt glycoprotein initially isolated by its ability to induce the expression of the enzyme 20 $\alpha$ steroid dehydrogenase in stem cells. However, this molecule has since been demonstrated to permit the survival, proliferation and development of progenitor cells from several distinct lineages including erythroid, granulocyte, macrophage, megakaryocytic and basophilic lineages and also to allow the self-renewal and differentiation of pluripotential stem cell populations. Studies of Interleukin 3 receptor binding to responsive cell lines reveals that there are 1500–2500 specific receptors per cell with a $K_D$ of 17 pM. No human equivalent which can compete with IL3 for occupation of this receptor has yet been found, but a protein which carries some similar properties was described by *M.A.S. Moore* (*Sloan–Kettering, USA*). This growth factor, named pluripoietin, has been purified to homogeneity from the conditioned medium of a human bladder carcinoma cell line (5637) and has a mol. wt of 18,000. Unlike Interleukin-3 this molecule was active across species barriers, stimulating murine granulocyte/macrophage and mixed colony formation, and stimulating the differentiation of the murine myelomonocytic cell line, WEHI-3BD$^+$ (as does Nicola's G-CSF, but not murine Interleukin-3). In addition pluripoietin acts, like (Interleukin-3), on primitive clonogenic progenitor cells (human CFU-GEMM) as well as on fully differentiated cells, for example, inducing protein synthesis in human monocytes and macrophages.

It was apparent from these contributions that a complex scheme involving both lineage restricted and multilineage haemopoietic growth factors is emerging. In addition some of these growth factors exhibit cross species reactivity, whilst others are unable to do so. Another important feature of these contributions was that the growth factors act at very low concentrations and in addition the receptor numbers per cell are extremely low. There is also intriguing evidence that very few receptors need be occupied to elicit a biological effect suggesting that a few molecules per cell can induce proliferation and differentiation.

### *Molecular cloning of haemopoietic growth factors*

Another route recently employed to obtain large quantities of pure haemopoietic growth factors is molecular cloning. A very clear account of cloning strategies was given by *Drs T. Yokota and F. Lee* (*DNAX, California*) who described the three important requisities as, firstly, a source of biologically active messenger RNA; secondly, a full length cDNA library in a mammalian expression vector; and thirdly reliable and sensitive biological assays. This strategy had been employed to good effect in their hands to clone murine Interleukin 3 and also Interleukin 2 (T cell growth factor). Yokota described Interleukin 3 as a polypeptide consisting of 134 amino acid residues, mol. wt 15,000. The discrepancy between this and Ihle's 28,000 mol. wt could be accounted for by the sugar groups attached to the protein at the 4 potential N-glycosylation sites, although the N-terminal sequence which Ihle has reported from an amino acid analysis of the purified protein appears to be several residues shorter than the reported sequence from the DNAX and Canberra groups (obtained via molecular cloning), an anomaly which is at present unexplained. Biological activities of Interleukin 3 produced by transfected cos-7 monkey cells were shown to include all the activities characteristic of Interleukin 3.

*A.R. Dunn* (*Ludwig Institute for Cancer Research, Melbourne*) has also installed full length cDNA fragments of not only Interleukin-3 but also granulocyte-macrophage colony stimulating factor (GM-CSF) into a eukaryotic expression vector and transfected into simian cos cells where they direct the expression of growth factors with apparently normal biological characteristics. For the GM-CSF molecule Dunn and co-workers (after having obtained the cDNA sequence using a strategy similar to that described by Yokota and Lee), have considered the nucleotide sequence at the 5′ end of GM-CSF messenger RNA. This reveals the presence of two AUG (initiation) codons associated with a stable loop structure both of which are in frame with the coding sequences of GM-CSF. Use of these 5′ AUG codons would result in a nascent polypeptide with the characteristics of a transmembrane protein, whereas use of the second would result in the synthesis of a hydrophobic leader sequence characteristic of a secreted protein. This gives rise to the intriguing speculation that GM-CSF may, in some cases, exist as a membrane bound protein which can present itself to neighbouring cells only. This may have important implications for the role of the stromal microenvironment in supporting haemopoiesis in that expression of growth factor activity at the membrane may play a key role in the cell:cell interactions, known to be of fundamental importance in haemopoiesis.

Using the sequences derived for GM-CSF and Interleukin 3, probes have been constructed (by both the DNAX and Melbourne groups) to identify a human counterpart to these murine molecules. However, both these groups have had little success in obtaining a human equivalent to Interleukin-3. On the other hand Lee (of DNAX) did report the identification of a human equivalent to murine GM-CSF whose biological activities were in the process of being tested.

Several important points arose from the work described by the molecular biologists. Firstly the molecular cloning of haemopoietic growth factors is possible and expression of biologically active growth factors can be achieved in both prokaryotic and eukaryotic systems. Second, it is apparent that the high degree of glycosylation found on many of these haemopoietic growth factors is not necessary for biological activity (this was reported by *A.D. Whetton* (*Paterson Laboratories, Manchester*), who said that studies by *L. Healey* and *G.W. Bazill* (*Paterson Laboratories, Manchester*) had shown that highly purified Interleukin 3, isolated from WEHI-3B cell conditioned medium, retained full biological activity when stripped of its carbohydrate groups with endoglycosidase F). Third, although some growth factors exhibit some species conservation this is not always the case. Fourth, production of large amounts of molecularly cloned material is at the moment presenting something of a problem. No effective procedure has yet been found to yield large amounts of biologically active protein from transfected eukaryotic or prokaryotic cells. Fifth, the structure and expression of the growth factor genes and the possible form the protein can take (i.e. membrane bound or soluble) can now be explored much more readily with the sequence data obtained by molecular biological techniques.

### *Biological activities of haemopoietic growth factors*

The range of activities and specificities of haemopoietic growth factors has been described to some extent but obviously many questions still remain. In particular how do all the regulatory molecules act together *in vivo* to maintain haemopoietic homeostasis and what are the events leading to disruption of haemopoiesis? Oncogenes have been implicated in growth regulation in many systems and their influence on the production of, and response to, haemopoietic growth factors is being investigated.

As in the murine and human systems, haemopoietic growth factors have been isolated from chickens. *A. Leutz* (*EMBL, Heidelberg*) described the purification of chicken myelomonocytic growth factor (cMGF), which has the ability to promote macrophage colony development from normal chicken bone marrow. This factor was also able to promote survival of cMGF-dependent chicken myeloblast cell lines that are produced following transformation by retroviruses encoding the *v-myb* and *v-myc* oncogenes. However, macrophages transformed by the MHZ virus were independent of cMGF for survival; the MHZ virus encodes both *v-myc* and the *src*-related *v-mil*. Leutz subsequently showed that the cMGF-dependent *v-myb* transformed myeloblasts could be rendered factor-independent by other *src*-related retroviral sequences (e.g. *v-fps, v-yes, v-ros*) but not by *v-myc*. Similarly, *v-src* converted the factor-dependent *v-myb* transformed cells to independence, but not the *v-myc* transformed myeloblasts. The conversion to factor-independence is associated with the production of a cMGF-like substance by the cells, indicating the the survival of the cells requires autocrine stimulation. Experiments along similar lines, with mouse cell lines, were described by Ihle. In this case IL3-dependent cell lines were infected with molecular recombinants containing *v-myc* from MHZ or MC-29 viruses, resulting in factor-independence of the cell lines. In further contrast to Leutz's results these factor-independent cells retained the receptors for IL3 but did not themselves produce it, nor did antiserum to IL3 block the growth of the cells. This suggests that the requirement for normal growth factor had been bypassed in some way.

Another approach to investigating the possible role of oncogenes in the control of haemopoiesis was discussed by *E. Spooncer* (*Paterson Laboratories, Manchester*). Long-term bone marrow cultures were infected with virus containing the *src* oncogene which resulted in long-term and stable changes in the haemopoietic activity in the cultures, *viz.* an almost complete block in the output of mature cell types and a build up of primitive cell types. This was accompanied by the expression of apparently unlimited self-renewal capacity by the haemopoietic stem cells. Furthermore, it was possible to derive cell lines from these cultures which were dependent on IL3 for their survival, were non-leukaemic and possessed many of the characteristics of the stem cell. The role of the oncogene in the transformation of haemopoietic activity following infection with *src* is not quite clear. Due to the failure to detect the *src* kinase activity specifically associated with cells of haemopoietic origin there is a possibility that these effects may have been mediated via a direct influence of the *src* oncogene on stromal cells of the haemopoietic environment, which subsequently resulted in a modification of haemopoietic activity. These studies showed diverse influences of v-oncogenes on haemopoiesis in promoting the emergence of factor-dependent cell lines; factor-independent cell lines which appear either to produce their own supply of IL3 or to be totally autonomous of normal growth regulation and in permitting extensive self-renewal of stem cells. This raises the question of whether analogous c-oncogenes are, in fact, involved in the control of normal haemopoietic cell development in response to growth factors.

Some progress has recently been made in describing, in biochemical terms, the nature of dependence of haemopoietic cells on IL3. Whetton demonstrated a chain of biochemical events which IL3-dependent cell lines undergo in response to IL3, but not in response to any other, non-specific, growth factors. In the presence of IL3, intracellular ATP levels are maintained in the cells. In its absence ATP levels rapidly fall. In fact limited survival of the cells could be effected by an ATP-regenerating system in the absence of IL3. The IL3 was shown to be acting on the ATP-generating machinery of the cell at the level of glycolysis and the point of regulation of this process is the transport of glucose into the cell by the glucose transport protein. Presumably the binding of IL3 to its receptor activates (directly or indirectly) the glucose transport mechanism and the subsequent production of ATP by glycolysis ensures survival of the cell. The requirement for some step in this chain of events is clearly bypassed in the case of the factor-independent cell lines described by Ihle and such cell lines should permit determination of the biochemical changes that lead to the emergence of autonomous haemopoietic cells.

In most studies of the action of growth factors the factors are produced *in vitro* from cell lines or normal tissues stimulated in some way. The *in vivo* function of the molecules and their source and presentation to target cells is poorly understood. Various suggestions indicate the production of growth factors by adherent (stromal) cells and accessory cells to the target cells. *J.W. Adamson* (*University of Washington, USA*) described a response of haemopoietic cells to PDGF, which was mediated by interaction of stromal cells with PDGF and a modulation of growth factor production from stromal cells (endothelial cells and fibroblasts) by other cell types, in this case, monocytes *C.J. Paige* (*Basel Institute for Immunology, Switzerland*) has developed a colony forming cell assay for B-cell progenitors which is dependent upon factors produced by accessory cells contained in the adherent foetal liver cell population. In fact IL3, M-CSF and GM-CSF have the capacity to promote the production of the factor required for development of the B-cell colonies from the accessory cell type. It is clearly more of a problem to perform studies on the *in vivo* relevance of growth factors in humans than mice. However, *H. Messner* (*Ontario Cancer Institute, Toronto, Canada*) has detected growth factors in platelet-free human plasma samples and in particular has correlated a prolonged presence of megakaryocyte-colony stimulating activity with a delayed return of platelets in patients following bone marrow transplantation.

*H. Broxmeyer* (*Indiana University, USA*) discussed a molecule termed leukaemia associated inhibitory activity which, by some criteria, may be classed as a member of the acidic isoferritin family. This molecule has the capacity to suppress colony formation by $Ia^+$ GM-CFC, BFU-E and MxCFC which are in cell cycle and from normal donors but not from leukaemic human donors. There is some difficulty, however, in fitting in a molecule with the characteristics described by Broxmeyer in any of the known categories of ferritins. The main objections, from *A. Jacobs* (*Welsh National School of Medicine, Cardiff, UK*) were the facts that the active concentration of this molecule on haemopoietic cells ($10^{-18}$M) is less than the plasma concentration of ferritin, that considerably more than this should be produced by the culture itself, and that a glysoylated acidic isoferrin (as described by Broxmeyer) has not previously been identified. According to Jacobs only serum ferritins are glycosylated and these molecules are not acidic in nature. Joint experiments between these groups so far yielded only variable and inconsistent results. Definite proof of the putative identify of the molecule which can suppress colony formation with acidic isoferritin appears to await assay of material expressed by the families of genes coding for subunits of acid isoferritins.

On a more general aspect of growth factors *M.F. Greaves* (*Leukaemic Research Fund, London, UK*) described the structure and role of the transferrin receptor. In discussions he clearly pointed out that it was important to be aware of the difference between more general growth factors, such as transferrin and its receptor which appear to function for all cell types, and lineage restricted specific growth factors which, in general, have been the subjects of this symposium. The transferrin receptor is present on all dividing cells, is increased in cycling cells and absent on quiescent cells. The transferrin receptor may also be involved in differentiation. Greaves discussed the possibility that NK cells may recognise the transferrin receptor and function as a cellular mechanism for downgrading the rate of growth. The fact that the expression of the transferrin receptor is ubiquitous on dividing cells, but the cellular targets for NK cells are more restricted suggests that this speculation must be somewhat refined in order to explain such a role for the transferrin receptor.

Overall, this symposium covered a wide range of aspects involved with the role of growth factors in haemopoiesis. Certainly tremendous advanced have been achieved since the 15th Paterson symposium on analysis of haemopoietic stem cell behaviour in 1980. Predictably, the advances have been particularly associated with use of recently developed techniques in molecular biology and clearly cell biologists and biochemists must now finish off defining the role of the growth factors, which will probably soon be available in a pure form and in large quantities. However, the step from *in vitro* studies to understanding the *in vivo* control of haemopoiesis still poses a big challenge.

The symposium was organised by *B.I. Lord* (*Paterson Laboratories, Manchester*), who assembled an excellent group of international scientists in this field, ensuring that discussion was lively and valuable. The Paterson Laboratories would like to acknowledge the sponsorship of the Cancer Research Campaign, UK, Searle Research and Development and ICI Pharmaceuticals Division. The CRC generously provided a grant for the travel of three overseas participants, Searle Research another and in a scheme to encourage the development of young scientists the CRC also sponsored the participation of two post-graduate students.